# Unraveling Crystal Phase-Driven Activity and Selectivity of WO_3_ for Photoelectrochemical Biomass Valorization

**DOI:** 10.1021/acs.inorgchem.4c05048

**Published:** 2025-01-13

**Authors:** Chin-Chan Wu, Truong-Giang Vo, Michael B. Sullivan, Khuong P. Ong, Hongmei Jin, Angela Chuang, Minh-Trang Huynh Pham, Chia-Ying Chiang

**Affiliations:** † Department of Chemical Engineering, National Taiwan University of Science and Technology, Taipei 10607, Taiwan; ‡ Institute of High Performance Computing, Agency of Science, Technology and Research (A*STAR), 1 Fusionopolis Way, Singapore 138632, Republic of Singapore; § Institute of Sustainability for Chemicals, Energy and Environment (ISCE^2^), Agency for Science, Technology and Research (A*STAR), 1 Pesek Road, Singapore 627833, Republic of Singapore

## Abstract

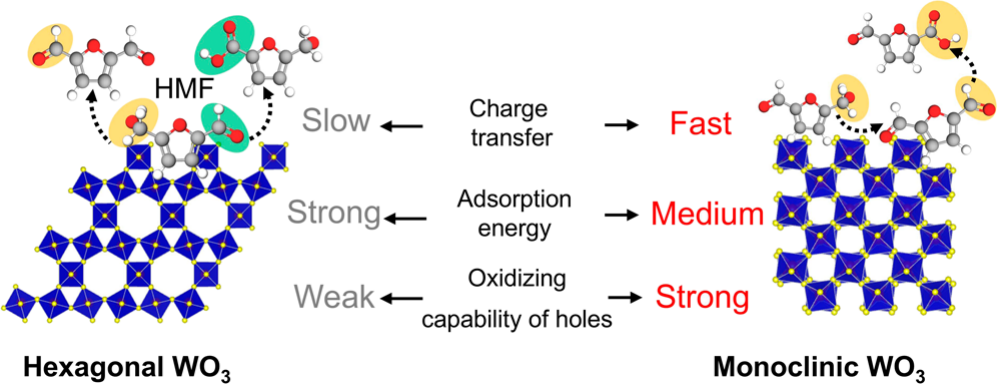

Modulating the crystal phase of a photocatalyst significantly impacts its surface and photochemical properties, allowing for the adjustment of catalytic activity and selectivity, particularly in the electrooxidation reactions of biomass-derived chemicals. Herein, monoclinic and hexagonal phases of WO_3_ are employed as photoanodes for the photoelectrochemical conversion of 5-hydroxymethylfurfural (HMF) to 2,5-diformylfuran (DFF). The monoclinic phase demonstrated exceptional performance in photoelectrocatalytic HMF oxidation, achieving remarkable photocurrent densities (1.1 mA cm^–2^), which were 5.5 times greater than those observed for hexagonal WO_3_. Moreover, the yield of DFF products obtained over monoclinic WO_3_ was approximately 2.5 times higher compared to that of hexagonal WO_3_. A combination of experiments and theoretical calculations indicates that the superior performance of monoclinic WO_3_ for HMF oxidation mainly originates from enhanced light harvesting efficiency, better charge separation and utilization, balanced adsorption energy, and stronger oxidative ability of photogenerated holes. This study emphasizes the potential of crystal phase engineering to regulate the reaction activity and selectivity and provides insights into how to design next-generation high-performance photoelectrodes for sustainable chemical production from biomass.

## Abstract 2

This study explores how the crystal phase of WO_3_ affects its photoelectrocatalytic performance in turning biomass-derived chemicals into valuable products. By comparing two phases of WO_3_, the monoclinic phase was found to outperform the hexagonal phase, producing more efficient and selective results in converting 5-hydroxymethylfurfural (HMF) into 2,5-diformylfuran (DFF). The findings highlight the role of crystal structure in improving light-based chemical processes, paving the way for better technologies in sustainable chemical production from biomass.

## 1 Introduction

In the face of rapid climate change and limited fossil fuel resources, research on sustainable energy sources and chemical production processes is gaining significant attention.^1^ Biomass, the most widely available renewable resource on Earth, is a potential feedstock for producing liquid fuels and chemicals.^2,3^ Of all biomass-derived chemicals, 5-hydroxymethylfurfural (HMF) stands out due to its synthetic versatility from cellulose and easy conversion to various valuable products.^2^ One particularly significant process is the electrochemical oxidation of HMF to yield either 2,5-furandicarboxylic acid (FDCA) or 2,5-diformylfuran (DFF).^1,4,5^ For instance, DFF is a strategic raw material for synthesizing a range of high-value polymeric materials and pharmaceutical chemicals, presenting expansive market opportunities. However, the targeted electrochemical transformation of HMF to DFF remains challenging due to low selectivity and a strong propensity for overoxidation.^6^

To date, most HMF oxidation reaction (HMFOR) studies focus on either photocatalytic^7−11^ or electrocatalytic processes,^12−17^ leaving a notable gap in the exploitation of a photoelectrochemical (PEC) method.^18^ Considering that PEC technology is an innovative approach that synergizes the merits of photocatalysis and electrocatalysis to enhance efficiency and reaction selectivity,^19^ it becomes essential to prioritize the development of a highly efficient PEC photoanode for HMFOR. Tungsten trioxide (WO_3_) has emerged as an appealing photoanode material for PEC applications, primarily because of its suitable band gap for visible light adsorption (2.6–2.8 eV), moderate hole diffusion length, high electron mobility,^20^ and reasonable stability under acidic conditions. Moreover, WO_3_ can crystallize in various phases, including orthorhombic, tetragonal, hexagonal, and monoclinic, each exhibiting distinct physical and chemical properties that can influence catalytic performance.^21−23^ For example, Yang et al. conducted a comparative analysis of the hydrogen evolution reaction activity in the monoclinic and hexagonal phases of WO_3_. It was revealed that between the two phases, monoclinic WO_3_ displayed superior catalytic performance owing to its lower charge transfer resistance and moderate hydrogen adsorption energy.^24^ In a separate study, Zhang et al. found that cubic WO_3_ exhibited a high methylene blue adsorption capacity, while monoclinic WO_3_ had exceptional photocatalytic activity for methylene removal.^25^ The progress of phase engineering underlines a potential direction to manipulate the catalytic activity by tailoring specific crystal phases of WO_3_, which modifies surface properties and charge transport.^26^ In the context of PEC for HMFOR, the molecule of HMF itself with its polar and organic functional groups can interact differently with distinct crystal phases; thus, reaction pathways and selectivity are varied. For instance, specific crystal phases may have higher surface reactivity, favorable adsorption, and activation of specific functional groups of HMF molecules, thereby directing the reaction toward a particular pathway and consequently impacting the product distribution. However, a comprehensive understanding of the complex interactions between the crystal phase and HMF is lacking, leaving a gap in the systematic manipulation of the WO_3_ crystal phase and their correlation with the activity and selectivity in PEC HMFOR.

Motivated by this knowledge gap, in this work, we conducted a systematic study of how crystal phase manipulation affects the PEC catalytic activity of WO_3_ photoanodes. Two stable crystal phases, hexagonal WO_3_ (h-WO_3_) and monoclinic WO_3_ (m-WO_3_), were selected as model crystal phases to explore their respective impacts on the PEC conversion of HMF to DFF. By shedding light on the mechanisms behind the phase-dependent catalytic performance of WO_3_, our study fills the gap in the literature and demonstrates the great promise of photoanode tailoring for sustainable biomass transformation.

## 2 Experimental Section

### 2.1 Preparation of WO_3_ with Different Crystal Phases

The WO_3_ films with different crystal phases were fabricated onto a fluorine-doped tin oxide (FTO, 15 Ω cm^–1^) glass substrate by the hydrothermal method and muffle furnace calcination as previously reported with slight modifications.^27^ Specifically, 1.0 g of (NH_4_)_2_WO_4_ was dissolved into 95 mL of Milli-Q water, followed by adding 3 mL of HCl (32%). Acidic conditions favor the formation of WO_3_ through controlled polymerization of tungstate ions and facilitate nanoplate formation.^28,29^ Afterward, 2 mL of H_2_O_2_ (30%) was added under continuous magnetic stirring until a transparent solution was obtained. The resulting mixture was transferred into a Teflon-lined stainless autoclave. A piece of FTO glass was immersed at a 45° angle, with the conductive side facing downward. The autoclave was sealed and heated at 160 °C for 4 h. Once cooled to room temperature, the substrate was removed, rinsed with deionized water, and air-dried at room temperature. The samples from hydrothermal processes were further annealed at different temperatures to induce the phase transition. The sample annealed at 350 °C was labeled h-WO_3_, whereas the sample annealed at 500 °C was denoted as m-WO_3_.

**Caution**: Hydrochloric acid (HCl, 32%) is corrosive. Hydrogen peroxide (H_2_O_2_) is irritative and possibly causes chemical and/or thermal burns. Appropriate protective measures should be employed during handling: goggles, masks, and rubber gloves must be worn.

### 2.2 Material Characterization

The morphology and microstructure of the catalyst were investigated by using a field emission scanning electron microscope (FESEM, JSM-6500F) and transmission electron microscopy (TEM, FEI Tecnai G2 F30). Crystal structures were characterized by using an X-ray diffraction spectrometer (XRD, G2 Bruker, Germany). Raman spectroscopy (MRID, Protrustech, Taiwan) with a 532 nm laser was used to study the structural and vibrational properties of the samples. The Raman spectra were acquired with a 10× objective lens, an acquisition time of 10 s, and at 15 accumulation, from 100 to 1000 cm^–1^. The surface elemental composition and oxidation states of the WO_3_ films were studied using X-ray photoelectron spectroscopy (XPS, VG ESCALAB 250) without special preparation. The peak position was calibrated using C 1s of 284.8 eV as a reference. The absorption profile was obtained using an ultraviolet–visible spectrophotometer (V-650, JASCO, Japan) with an integrating sphere accessory. Diffuse reflectance spectra were measured over the 200 to 800 nm wavelength range. A blank FTO substrate was used as a baseline. All crystal structures were retrieved from the Material Project open database source, while atomic visualizations were produced using VESTA.^30^

### 2.3 Photoelectrochemical Performance Evaluation

Photoelectrochemical HMFOR performance was evaluated in a custom-made H-shaped quartz cell with a three-electrode configuration. The working electrode was WO_3_-deposited FTO with (an exposed surface area of 1 cm^2^), with a carbon rod serving as a counter electrode and an Ag/AgCl (in 3 M KCl) as the reference electrode. A 500 W Xe lamp (Newport) coupled with an AM 1.5G filter was used as the light source. The light intensity was set at 100 mW cm^–2^ using a thermopile sensor (919P, Newport) with a power meter (843-R, Newport). Aqueous sodium tetraborate solution (NaB_i_, 0.1 M) with 5 mM HMF, adjusted to pH 4 using 2 M H_2_SO_4_, was used as an electrolyte, unless otherwise specified. The electrochemical parameters were controlled using an Autolab potentiostat (PGSTAT204, Netherlands), with all potentials referred to a reversible hydrogen electrode (RHE) using the Nernst equation *E*_(RHE)_ = *E*_(Ag/AgCl)_ + 0.21 + 0.059pH. Electrochemical impedance spectroscopy (EIS) spectra were performed at 1.1 V_RHE_ across a frequency range from 10^5^ to 10^–2^ Hz under AM 1.5G irradiation. The Mott–Schottky (M–S) curves were measured at 10 mV/s and 1000 Hz under dark conditions. Incident-photon-to-current conversion efficiency (IPCE) was measured at different incident light wavelengths ranging from 300 to 500 nm, using a 500 W xenon arc lamp paired with a monochromator (Oriel Cornerstone, Newport). The investigation involved chronoamperometric experiments over 3 h, wherein potentials ranging from 0.7 to 1.1 V were systematically applied.

### 2.4 Product Analysis

At hourly intervals during the reaction, 50 μL of the electrolyte was withdrawn, diluted with 20 mM H_3_PO_4_ with a dilution factor of 20, and subsequently filtered through a 0.22 μm filter to remove any potential contaminants. After that, 20 μL of the filtered sample was subjected to high-performance liquid chromatography (HPLC, Young Lin, Korea) with an ultraviolet detector (284 nm) and a CarboSep CHO 87C column. The mobile phase was 20 mM H_3_PO_4_ at a flow rate of 0.5 mL/min. Details on the calculations are available in Supporting Information.

## 3 Results and Discussion

**Figure 1:**
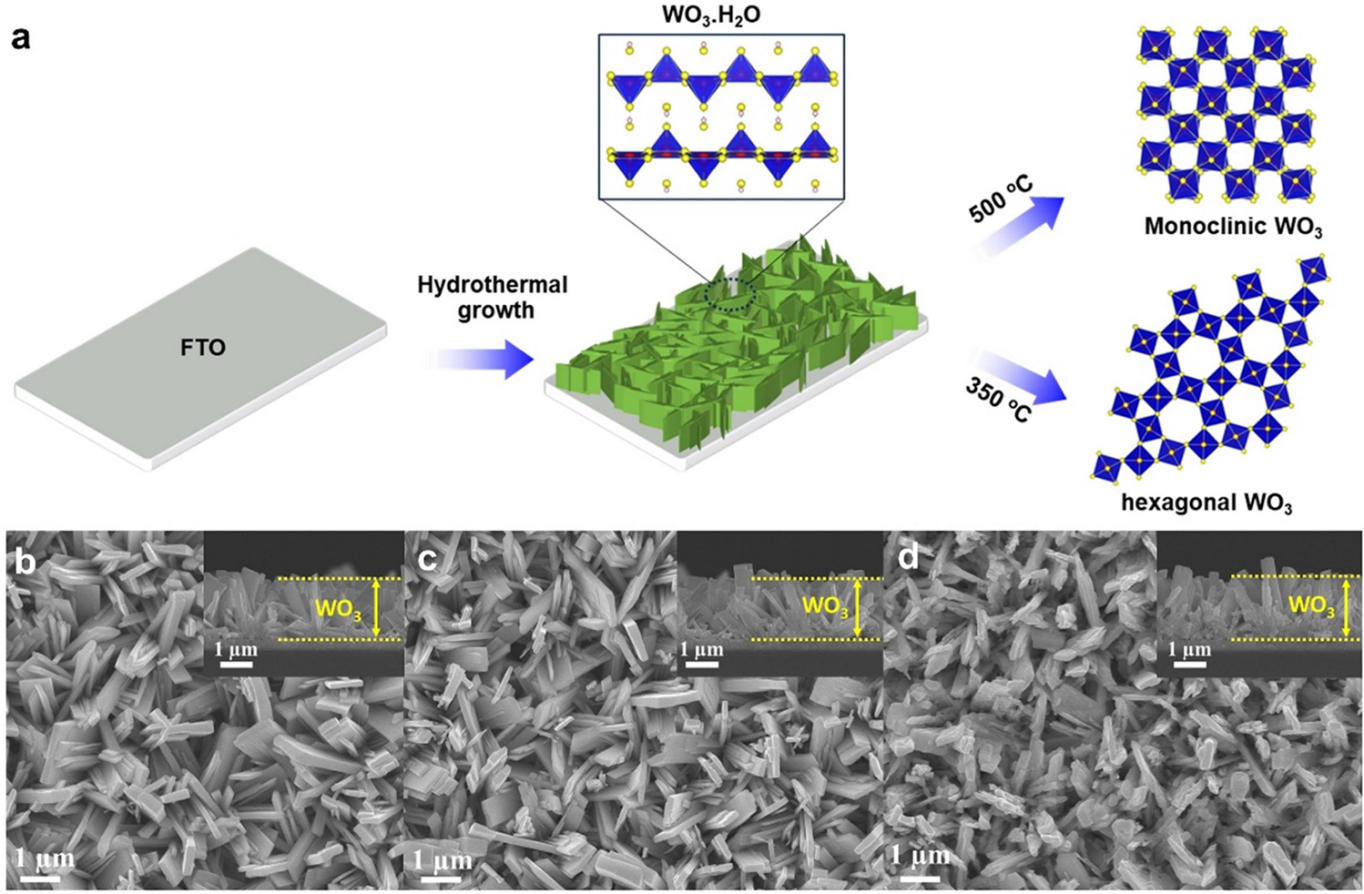
(a) Schematic illustration for preparing hexagonal and monoclinic WO_3_, (b–d) SEM images of as-prepared WO_3_·0.33H_2_O, h-WO_3_, and m-WO_3_. The insets show the corresponding cross-sectional SEM images.

To facilitate the investigation of phase-dependent photoelectrochemical activity and selectivity without introducing other external factors such as particle size change or composition,^31,32^ WO_3_ photoanodes with different crystal phases were synthesized on fluorine-doped tin oxide (FTO) glass substrates using a consistent hydrothermal method, altering only the annealing temperatures (Figure 1a). The microscopic structures of the materials prepared at different annealing temperatures were examined using FESEM. As shown in Figure 1b, the as-prepared WO_3_ exhibited a uniform coverage of distinctive plate-like structures, with thickness ranging from 30 to 70 nm, and tended to pack with adjacent nanoplates and oriented perpendicularly to the FTO substrate.^33^ The plate-like shape was maintained during the calcination process up to 350 °C (Figure 1c). As the annealing temperature increased to 500 °C, the WO_3_ nanoplates exhibited more pronounced cracking compared to the as-prepared one due to the elimination of water between the tungstite layers.

**Figure 2:**
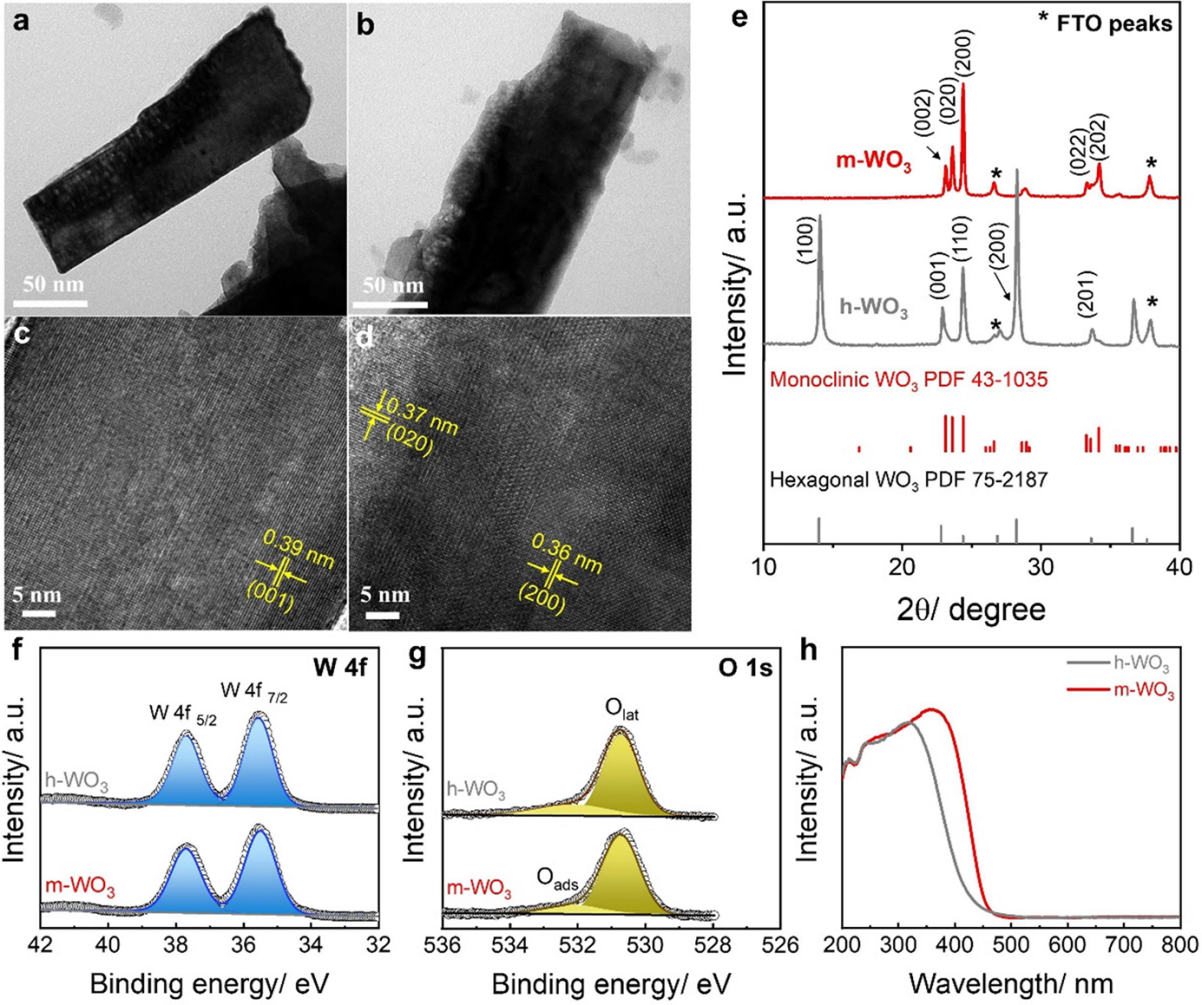
TEM images and HR-TEM images of (a, c) h-WO_3_ and (b, d) m-WO_3;_ (e) XRD patterns of h-WO_3_ and m-WO_3_; High-resolution XPS spectra for (f) Bi 4f and (g) O 1s and (h) UV–vis absorption spectra of h-WO_3_ and m-WO_3_.

The low-magnification TEM images in Figure 2a, b showed that both h-WO_3_ and m-WO_3_ exhibit a plate-like morphology, with dimensions of 50–80 nm in width and approximately 200–300 nm in length. The high-resolution TEM image of h-WO_3_ showed clear lattice fringes with a *d*-spacing of 0.39 nm, corresponding to the (001) crystal plane. In the case of m-WO_3_, ordered lattice fringes with spacings of 0.36 and 0.37 nm, corresponding to the (200) and (020) planes of monoclinic WO_3_, were observed. The XRD was subsequently performed to examine the crystal structures of hydrothermally as-prepared and those subjected to annealing at different temperatures. The XRD patterns of the unannealed, as-grown WO_3_ were matched well with the orthorhombic tungsten oxide hydrate (WO_3_·0.33H_2_O) phase (JCPDS 72–0199) (Figure S1). At relatively low annealing temperatures (<300 °C), a mixture of orthorhombic WO_3_·H_2_O and hexagonal WO_3_ (JCPDS 75-2187) was obtained (Figure 2e). A pure hexagonal phase was obtained at 350 °C, while a further rise in the annealing temperature to 500 °C resulted in a transition to the monoclinic phase (JCPDS 43-1035), illustrating temperature-induced crystal reconstruction.^27^

Raman spectra, as shown in Figure S2, exhibited distinct characteristic peaks associated with W–O–W bond vibrational modes. A noteworthy red shift was observed, indicating changes in the original coordination environment, specifically alterations in the W–O–W bond length, likely due to structural modifications during the thermal-induced phase transition from hexagonal to monoclinic.^34,35^ The high-resolution XPS spectra of W 4f and O 1s show a consistent position and comparable shapes for both h-WO_3_ and m-WO_3_, indicating the similarity in the surface composition between the two samples, characterized by the presence of W^6+^, lattice oxygen (O_latt_), and surface-adsorbed oxygen (O_ads_) (Figure 2f,g). In addition, UV–vis absorption spectra indicated that m-WO_3_ demonstrated higher visible light adsorption than h-WO_3_ absorption, with a redshift in absorption threshold from 430 nm (h-WO_3_) to 470 nm (m-WO_3_). The band gaps estimated from the Tauc plot (Figure S3) of h-WO_3_ and m-WO_3_ were 2.88 and 2.72 eV, highlighting the influence of the crystal phase on the optical properties. The higher annealing temperature employed in obtaining m-WO_3_ may also have contributed to improved film crystallinity, resulting in a gradual decrease of the energy band gap, which agrees with the results reported by other groups.^36,37^

**Figure 3:**
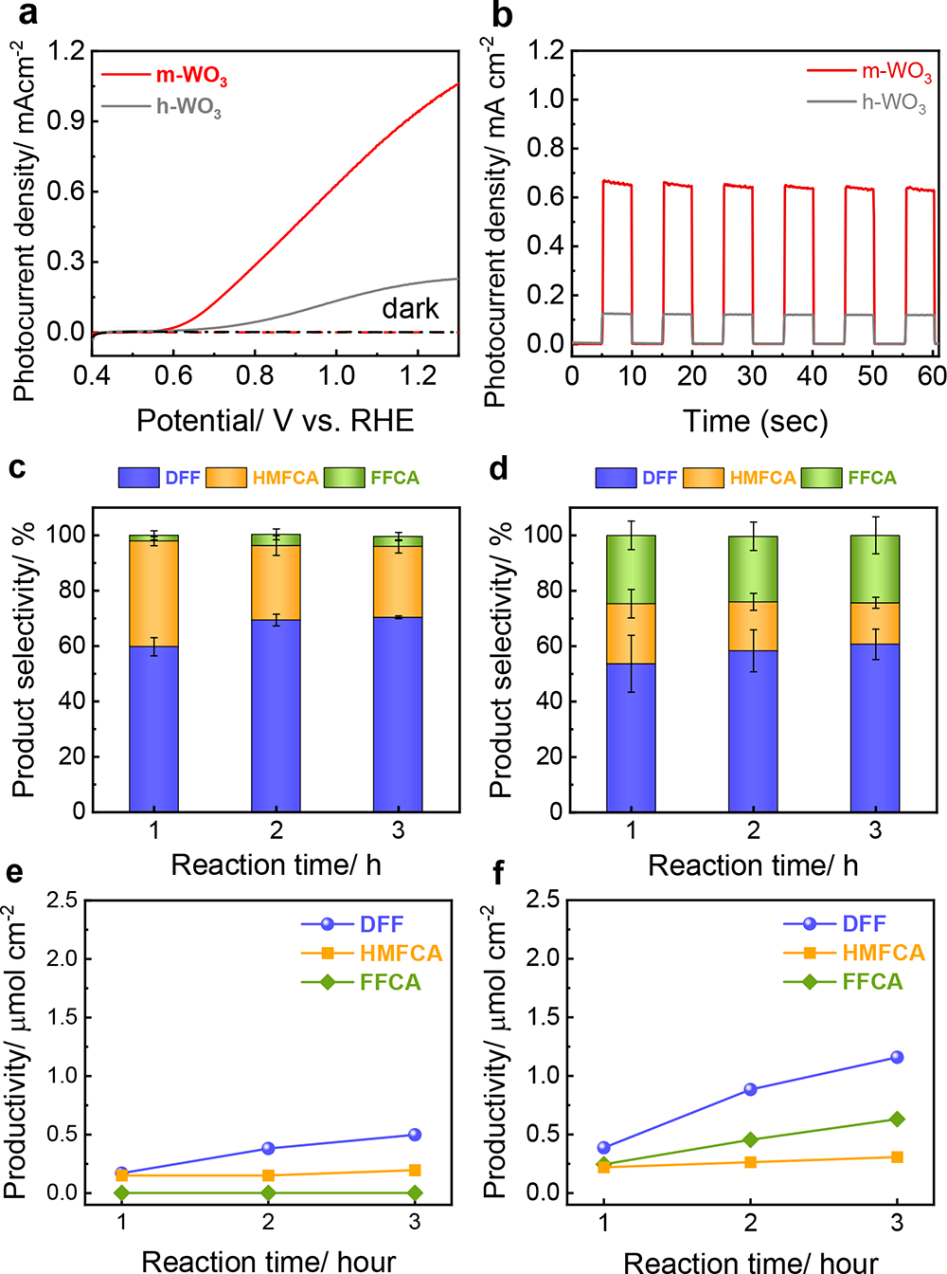
Photoelectrochemical HMFOR performance characterization of h-WO_3_ and m-WO_3_: (a) LSV curves; (b) transient *I*–*t* curves, (c, d) time-evolution product distribution and (e, f) productivity of h-WO_3_ and m-WO_3_ at 1.1 V vs RHE in 0.1 M NaB_i_ with 5 mM HMF under AM 1.5G irradiation, respectively.

The successful modulation of WO_3_ crystal phases has facilitated a meticulous exploration of how the crystal phase variations impact the performance of PEC HMFOR. Our investigation involved comparing the HMFOR efficacy of both h-WO_3_ and m-WO_3_ photoanodes in a 0.1 M NaB_i_ electrolyte containing 5 mM HMF, using a conventional three-electrode configuration. As shown in Figure 3a, the photocurrent-response profile revealed a striking disparity in photocurrent density, with m-WO_3_ significantly outperforming h-WO_3_. Specifically, the photocurrent on h-WO_3_ started to rise at 0.7 V_RHE,_ and a photocurrent density of only 0.20 mA/cm^2^ was obtained at 1.3 V_RHE_. Meanwhile, m-WO_3_ exhibited a slightly lower onset potential at 0.6 V_RHE_ and a rapid increase in photocurrent, reaching 1.1 mA/cm^2^ at 1.3 V_RHE_, resulting in a nearly 6-fold enhancement.

To evaluate the stability and instant photoresponse of both samples, *I*–*t* curves were recorded at 1.1 V_RHE_ under both intermittent and continuous light irradiation, as shown in Figure 3b. As shown in Figure 3b, it was observed that the photocurrent remained constant throughout the experiment and the recorded photocurrent densities matched those obtained from the LSV measurements (Figure 3a). Upon the light was switched off, the photocurrent of both h-WO_3_ and m-WO_3_ quickly dropped to nearly zero. When the light was switched on again, photocurrents were promptly regained without any delay, suggesting their good photoresponse and photoconductivity. Meanwhile, under constant irradiation at an applied potential of 1.1 V_RHE_, the time-evolution photocurrent profiles show relatively good durability, with approximately 50% of the initial photocurrent remaining for 3 h (Figure S4a). Additionally, postreaction characterization showed no phase transition, dissolution, or chemical state changes for either sample (Figure S5), demonstrating their reasonable photochemical stability for PEC HMFOR.

High-performance liquid chromatography (HPLC) analysis of PEC HMFOR products obtained over h-WO_3_ and m-WO_3_ photoanodes at 1.1 V is presented in Figure S6. Although with different dominant compositions, diformylfurfural (DFF), 5-Hydroxymethyl-2-furancarboxylic acid (HMFCA), and 5-formylfurancarboxylic acid (FFCA) were observed in the products of both samples. Figure 3c, d presents the distribution of HMFOR products over h-WO_3_ and m-WO_3_ photoanodes. Of significance, DFF was the dominant product, which accounted for approximately 55 and 70% of h-WO_3_ and m-WO_3_, respectively. The DFF selectivity in this study is among the highest results reported in the literature for PEC HMFOR (Table S1). The second primary product for m-WO_3_ is FFCA, which is produced from the further oxidation of either HMFCA or DFF. In contrast, HMFCA was predominantly yielded as the secondary major product from h-WO_3_. This observation indicates distinct reaction mechanisms and oxidation levels that might have occurred over the two photoanodes and will be discussed in more detail in a later section. Figure 3e,f illustrates the temporal evolution of the detected reaction products, which show an increase with reaction time. After a 3 h PEC HMFOR, the amount of high-value products, particularly DFF, generated over m-WO_3_ was more than 2.5-fold higher than those obtained over h-WO_3_.

**Figure 4:**
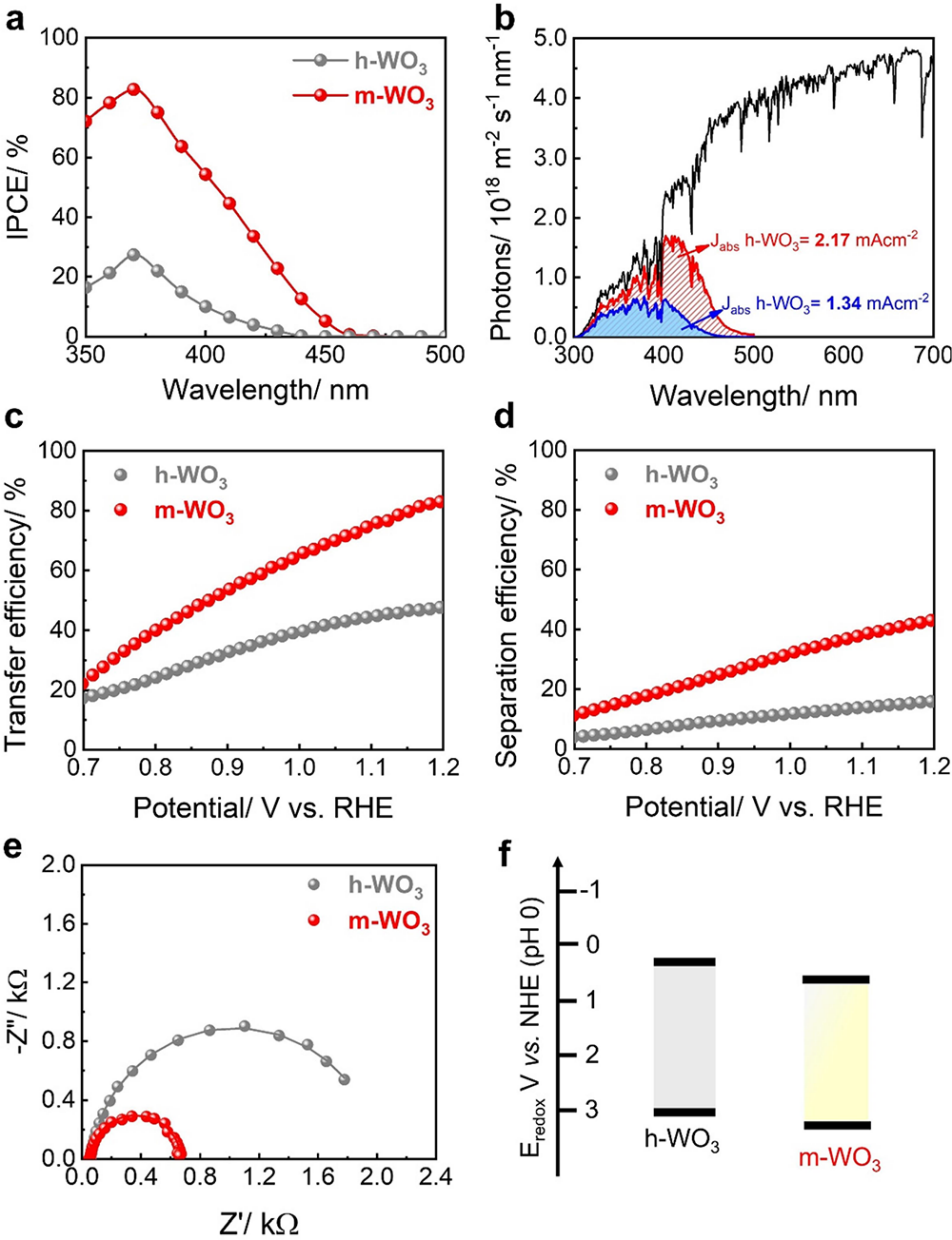
(a) IPCE of h-WO_3_ and m-WO_3_ at 1.1 V in 0.1 M Na_2_B_4_O_7_ + 5 mM HMF (pH 4) solution under AM 1.5 G irradiation; (b) photon flux under AM 1.5 G (ASTM G173-03) and those weighted by the absorption spectra of h-WO_3_ and m-WO_3_ films; (c) hole injection and (d) separation efficiency of h-WO_3_ and m-WO_3_ in 0.1 M Na_2_B_4_O_7_ + 5 mM HMF (pH 4) solution under AM 1.5 G irradiation; (e) Nyquist plot of h-WO_3_ and m-WO_3_ at 1.1 V in 0.1 M Na_2_B_4_O_7_ + 5 mM HMF (pH 4) solution under AM 1.5 G irradiation. (f) Tentative band position of h-WO_3_ and m-WO_3_.

For typical PEC reactions, the overall performance is also closely linked to the efficacy of electron–hole generation, separation, and utilization; specifically, how effectively photogenerated holes participate in the reactions. Therefore, the incident photon-to-current efficiency (IPCE) of the h-WO_3_ and m-WO_3_ photoanodes was first measured at an applied bias voltage of 1.1 V_RHE_ (Figure 4a)**.** A higher IPCE for the whole range of wavelengths is observed for m-WO_3_ with a maximum of 80% at 370 nm. This improvement can be attributed to better light absorption or charge separation. The electron flux density analysis (Figure 4b) reveals that m-WO_3_ exhibits a significantly greater optical response capability compared to h-WO_3_. This is reflected in the higher theoretical current density (2.17 vs 1.34 mA, assuming an APCE of 100%), indicating that the superior optical properties of m-WO_3_ are among the contributing factors to the observed differences in PEC performance. To assess the inherent efficiency of the materials, absorbed photon-to-current conversion efficiency (APCE), which reveals the internal conversion capability of photoanodes,^38^ was also calculated. As depicted in Figure S7, the APCE of m-WO_3_ was observed to be about 80–90% in the range 350–380 nm, which is more than twice that of h-WO_3_, indicating the better charge separation properties of m-WO_3_. To further corroborate this conclusion, interfacial hole-injection efficiency (η_inj_) and bulk charge separation efficiency (η_sep_) for the PEC HMFOR on h-WO_3_ and m-WO_3_ were also investigated (see ESI and Figure S8 for details). As shown in Figure 4c, m-WO_3_ exhibits a superior interfacial hole injection rate than h-WO_3_ across the whole potential window, achieving 74% at 1.1 V_RHE_, which confirms the enhancement of the kinetics of HMF oxidation of m-WO_3_ (Figure 4d). The η_sep_ of m-WO_3_ was also more than twice that of h-WO_3_. The electrochemical impedance spectroscopy (EIS) analysis was applied to reveal charge transfer kinetics at the electrode–electrolyte interface. Generally, the smaller the diameter of the semicircle, the better interfacial charge separation and transfer.^39^ As shown in Figure 4e, the h-WO_3_ sample with pure hexagonal phase exhibited a larger semicircle than m-WO_3_, indicating inferior charge transfer efficiency of the hexagonal phase compared to the monoclinic phase and thus lower photocurrent. The Mott–Schottky (M–S) analysis, which can provide information about the charge carrier concentration of both samples, is shown in Figure S9. Surprisingly, h-WO_3_ showed smaller slopes than did m-WO_3_, suggesting a higher concentration of carriers. On the one hand, h-WO_3_ displayed a high carrier concentration, while on the other hand, it exhibited inferior performance in terms of current density and product yield. Such duality might stem from multiple contributing factors apart from carrier concentration, which involves charge injection efficiency and band edge positions in governing the PEC performance of photoanodes. Indeed, overall PEC performance is not solely dependent on carrier concentration but also on how efficiently the photogenerated holes are separated and utilized. If charge carriers cannot be effectively consumed by the reactant molecules due to slow kinetics, they are prone to recombination, leading to inferior performance. In that case, the electronic properties derived from the Mott–Schottky analysis may not fully correlate with others, where kinetics, interface chemistry, and band position are involved. Different band edge positions between the two phases could be another significant factor influencing the activity and selectivity of PEC HMFOR. A photoanode with a well-matched valence band (VB) compared to the other, would show improved oxidation activity even at lower carrier concentration. In fact, as depicted in Figure 4f, the VB of m-WO_3_ was more positive than those of h-WO_3_. This suggests a greater oxidation capability for holes,^40,41^ allowing holes to efficiently oxidize the HMF molecules, resulting in higher current densities.

Above, we have discussed the possible underlying reasons for the higher photocurrent and productivity of m-WO_3_ compared to h-WO_3_. Yet, it remains unclear why there is a difference in selectivity between these two phases. Before further delving into this interesting aspect, it is necessary to briefly recall the reaction pathway and potential products in the PEC HMFOR. As shown in Figure S10, HMF is initially oxidized to DFF or HMFCA. Further oxidation reactions then yield FFCA and, finally, FDCA.^17,42−46^ Notably, FFCA can be produced from the oxidation of HMFCA, DFF, or both. If FFCA originated from the HMFCA oxidation, this indicates that HMF-to-DFF and HMF-to-HMFCA are equally favored on m-WO_3_ with a selectivity of approximately 50% for each. Alternatively, one can also argue that FFCA is from DFF oxidation. If that is the case, both h-WO_3_ and m-WO_3_ favor the oxidation of hydroxyl groups of HMF to produce DFF, but somehow, DFF is easier to get further oxidized to FFCA on m-WO_3_ but not on h-WO_3_. To investigate this, we conducted oxidation experiments with HMFCA and DFF over m-WO_3_ under conditions identical to those used for HMF oxidation, except that either HMFCA or DFF (5 mM) was added to the electrolyte instead of HMF. As shown in Figure S11, FFCA was detected from both HMFCA and DFF oxidation, but the latter reaction produced much more FFCA with higher conversion than the former, suggesting that the DFF oxidation to FFCA is more favorable than HMFCA oxidation. Thus, it can be safe to conclude that on m-WO_3_, FFCA originates from DFF oxidation rather than HMFCA oxidation. It should also be noted that this does not completely rule out that contribution from HMFCA oxidation, albeit with a much lower probability and extent.

The next question is why DFF underwent further oxidation on m-WO_3_ but not on h-WO_3_. As previously discussed, one possibility for these differences in oxidation preference could be the oxidation ability of photogenerated holes, which again depends on the band edge positions. The stronger the hole oxidation activity, the more likely it is that further oxidation will occur. To test this hypothesis, we performed HMFOR at different applied biases. It was found that regardless of applied potentials from 0.7 V (just right after onset potential) to 1.1 V (a much higher overpotential), a constant selectivity of 15% for HMFCA was observed (Figure S12). Meanwhile, the higher potential or driving force resulted in lower DFF selectivity and higher FFCA selectivity, supporting the assumption of stronger hole oxidation activity and facilitating further oxidation.

**Figure 5:**
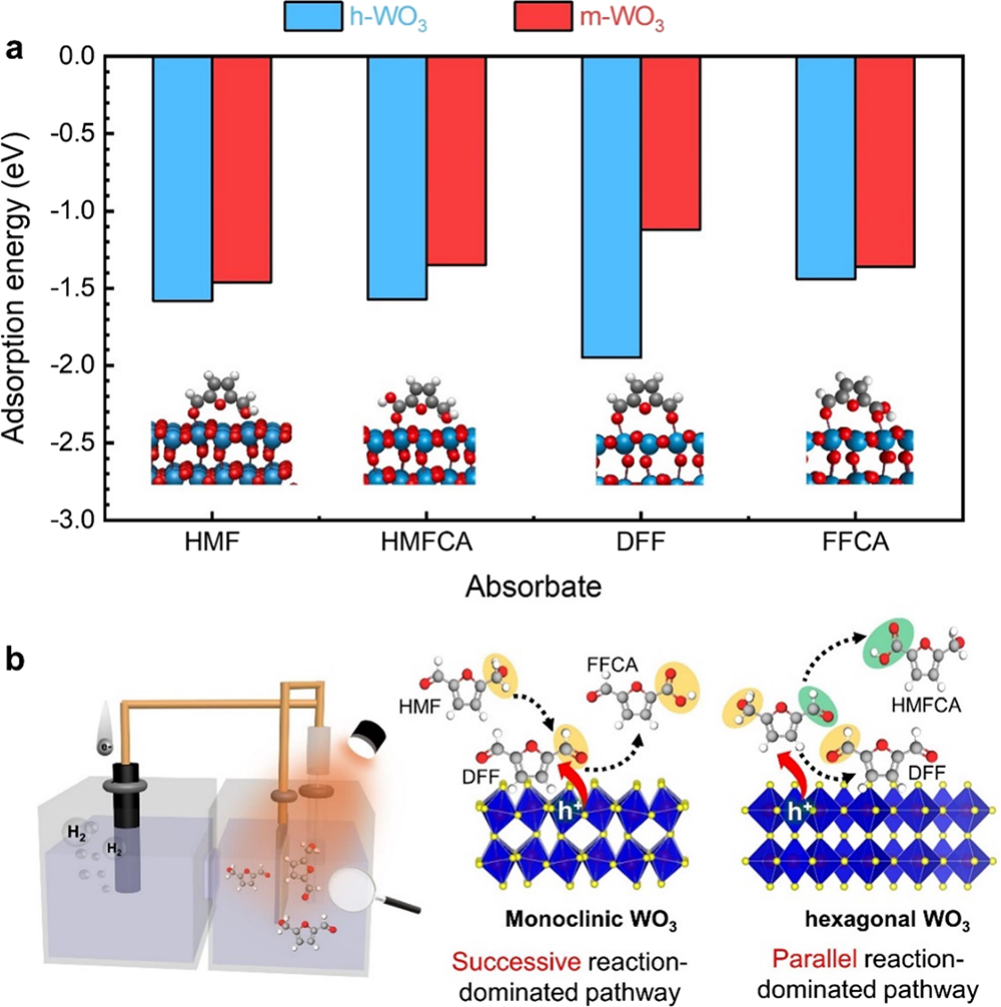
(a) Adsorption energies of HMF and its oxidized products on the (001) surface of h-WO_3_ and m-WO_3_. The inset shows the most stable adsorption configurations; (b) illustration demonstrating the tentative processes and mechanism over h-WO_3_ and m-WO_3_.

Another reasonable hypothesis is the effect of the phase-dependent reactant adsorption behavior. Different crystal phases with distinct atomic arrangements can change the adsorption energies between surface and adsorbates, leading to different production distributions.^47^ DFT calculations were then performed to explore the phase-dependent adsorption on the m-WO_3_ and h-WO_3_ surfaces. The models of HMF and its oxidized products (HMFCA, DFF, FFCA) adsorbed on the (001) plane of h-WO_3_ are shown in Figure S13, while the absorption energies (*E*_ad_) of the most stable configuration are shown in Figure 5a. The *E*_ad_ values of HMF on h-WO_3_ and m-WO_3_ were found to be −1.58 and −1.46 eV, respectively. This indicates a stronger binding affinity of HMF to the hexagonal WO_3_ surface than to the monoclinic surface. The slightly lower adsorption energy of m-WO_3_ reduced the reaction energy barrier,^48−50^ allowing HMF to participate in the reaction more easily, thus the higher PEC activity. This is consistent with the previous research, which stated that the electrocatalytic activity of HMFOR initially improved but then decreased as the *E*_ad_ values increased.^49,51^ More importantly, h-WO_3_ also binds DFF stronger than m-WO_3_ and even significantly more strongly than it does bind HMF. While prolonged adsorption of the DFF intermediate might be expected to facilitate its oxidation to the subsequent oxidized product, it is noteworthy that less than 5% of FFCA was detected over h-WO_3_. This suggests that the high adsorption energy of DFF on h-WO_3_ likely leads to a correspondingly high energy barrier for its further oxidation. Consequently, we postulate that h-WO_3_ is nearly inactive toward DFF oxidation due to this combination of strong adsorption and high activation energy requirements. Meanwhile, DFF with moderate adsorption energy, coupled with a higher oxidation capacity of the photogenerated holes on m-WO_3_, may be further oxidized to the subsequent product, FFCA (Figure 5b). This hypothesis explains the different product distributions observed.

## 4 Conclusions

Overall, the successful modulation of WO_3_ crystal phases enabled a comprehensive study of their impact on the PEC HMFOR performance. Monoclinic WO_3_ demonstrated superior photocurrent response, stability, and product yield compared to hexagonal WO_3_, primarily due to enhanced light absorption, better charge separation, and efficient reactant adsorption. It is postulated that, in addition to crystal phase manipulation, carefully tuning the band position and adsorption energy of HMF and its oxidized intermediates on the photoanode could potentially control both the activity and the selectivity of the reaction. These findings provide critical insights for optimizing the photoelectrochemical (PEC) performance of photoanodes in solar-driven chemical conversions.

## Supporting Information Available

Additional experimental, calculation, method details, Raman spectra, Tauc plot, stability data, Mott–Schottky, HPLC chromatograms, product analysis, and details of the density functional theory (DFT) calculations and comparison table (PDF)
