# Supplementary material for: Unraveling Crystal Phase-Driven Activity and Selectivity of WO3 for Photoelectrochemical Biomass Valorization
Source: Inorg Chem. 2025 Jan 13;64(3):1579–86. doi: 10.1021/acs.inorgchem.4c05048 (PMC11776042; doi:10.1021/acs.inorgchem.4c05048)
Supplement: Supplementary file 1 — ic4c05048_si_001.pdf [file ic4c05048_si_001.pdf]

Supporting information

Unraveling Crystal Phase-Driven Activity and Selectivity of WO_3 for Photoelectrochemical Biomass Valorization

Chin-Chan Wu[1]†, Truong-Giang Vo[2]†, Michael B. Sullivan[3], Khuong P. Ong[3], Hongmei Jin[3], Angela Chuang[1], Minh-Trang Huynh Pham[1], Chia-Ying Chiang[1]**

[1] Department of Chemical Engineering, National Taiwan University of Science and Technology, Taipei 10607, Taiwan

[2] Institute of Sustainability for Chemicals, Energy and Environment (ISCE[2]), Agency for Science, Technology and Research (A*STAR), 1 Pesek Road, Singapore 627833, Republic of Singapore

[3] Institute of High-Performance Computing, Agency of Science, Technology and Research (A*STAR), 1 Fusionopolis Way, Singapore 138632, Republic of Singapore

† *These authors contributed equally to this work*

Corresponding Authors:

Prof. Chia-Ying Chiang. Email: cychiang@mail.ntust.edu.tw

Dr. Truong-Giang Vo. Email: vincent_vo@isce2.a-star.edu.sg

Section 1 Detailed calculations

Calculation of Tauc's plot

Band gap energy was determined from the UV-vis spectra using the Tauc equation.

$$\alpha h\upsilon = A(h\upsilon - E_g)^n \quad \text{(S1)}$$

where α, h, υ, A, and E_g represent the absorption coefficient, Planck constant, light frequency, a constant, and bandgap energy. The coefficient n depends on the optical transition type of the semiconductor and n = 2 for WO_3.

Calculation of product selectivity

The selectivity (%) of each product was calculated based on the following equation

$$\text{Selectivity } \% = \text{specific product (mole)/ all product (mole)} \times 100\% \quad \text{(S2)}$$

Calculation of Incident photon-to-current efficiency (IPCE)

IPCE values were calculated using the following equation:

$$\text{IPCE } (\%) = \frac{J \times 1240}{\lambda \times P_{light}} \times 100\% \quad \text{(S3)}$$

where J, λ, and Plight are the photocurrent density (mA cm^{-2}), the wavelength of the incident light (nm), and the power density obtained at a specific wavelength (mW cm^{-2}), respectively.

Photocurrent density estimation from photon absorption rate

The absorption rate of photons, known as Jabs, is calculated by integrating the overlapped area between the absorption spectrum of the sample and an AM 1.5G solar irradiance.

$$J_{abs} = e \int_{\lambda_1}^{\lambda_{abs}} LHE \frac{P(\lambda)}{h\upsilon} d\lambda \quad \text{(S4)}$$

where $LHE = 1 - 10^{-A}$, A is the absorbance according to the UV-vis spectrum, P(λ) emitted power per surface, e (C) is the elemental charge and λ is the wavelength of the incident photon.

Calculation of hole injection efficiency

The solar-driven photocurrent (J_{abs}) undergoes two significant losses of charge carrier recombination in bulk and at the interface. Hence, the measured photocurrent during a typical reaction is expressed as follows:

$$J = J_{abs} \times \eta_b \times \eta_i \quad \text{(S5)}$$

In the presence of a hole scavenger, such as Na_2SO_3, it can be assumed that all holes reaching the electrode surface can be effectively consumed ($\eta_{i,\ SO_3^{2-}}=1$), thereby allowing the calculation of charge separation and transfer (also known as hole injection) efficiencies as follows:

$$\eta_b = J_{SO_3^{2-}}/J_{abs} \quad \text{(S6)}$$

$$\eta_i = J_{H_2O}/J_{SO_3^{2-}} \quad \text{(S7)}$$

Estimation of conduction band (CB) and valence band (VB)

The conduction band (CB) and valence band (VB) edges of WO_3, an n-type semiconductor, were determined by combining UV-Vis spectroscopy and Mott-Schottky analysis. The optical bandgap energy (E_g) was calculated from the Tauc plot derived from UV-Vis absorbance data. The Mott-Schottky analysis provided the flat-band potential (E_{fb}) relative to the reference electrode. Assuming the flat-band potential approximately corresponds to the position of the CB edge for n-type semiconductors, the VB edge was then calculated as $E_{VB}=E_{CB}-E_g$.[1,2]

---

[1] Beranek, Radim, (Photo)electrochemical Methods for the Determination of the Band Edge Positions of TiO_2-Based Nanomaterials, *Advances in Physical Chemistry*, 2011, 786759, 20 pages, 2011.
[2] Chen, M., Dong, H., Xue, M. *et al.* Faradaic junction and isoenergetic charge transfer mechanism on semiconductor/semiconductor interfaces. *Nat Commun*, 2021, **12**, 6363.

Section 2 Details on DFT calculation

All calculations were performed using the Vienna Ab initio Simulation Package (VASP). [3] [4] Core electronic states were represented with projector augmented-wave (PAW) pseudopotentials [5] [6] and the valence electronic states were expanded in terms of a planewave basis set with an energy cutoff of 700 eV. To account for exchange and correlation effects, the Perdew−Burke−Ernzerhof (PBE) functional was employed.[7] Since rather large molecules are involved, we used the DFT-D3(BJ)) method[8] [9] to account for van der Waals effects and, as such, all the calculations were performed at the Γ-point. The h-WO_3 and m-WO_3 surfaces were built as 2x2 supercells and contained 4 layers with the bottom two layers frozen. The length of the vectors for h-WO_3 was 14.596 Å x 14.596 Å x 30.000 Å and 15.832 Å x 15.473 Å x 30.00 Å for the m-WO3. This allowed a vacuum of at least 12 Å to avoid interactions between adsorbates and slab images. We used our internal software for surface workflows called *CatPlat*[10] to screen the initial adsorption of HMF on the WO_3 surfaces and identify the strongest adsorption energy; we tested 43 HMF configurations for h-WO_3 and 60 configurations for m-WO_3. Once the HMF configuration was selected, it was used for further studies of other molecules. The adsorption energies were calculated as :

Adsorption energy $E_{ads} = E_{slab+M} – E_{slab} - E_{M}$

where E_{slab+M} is the total energy of the slab with adsorbate, E_{slab} is the energy of the clean slab, and E_{M} is the energy of the isolated adsorbate molecule.

---

[3] Kresse, G.; Hafner, J. Liquid-Metal Amorphous-Semiconductor. Phys. Rev. B 1994, 49, 14251−14269. (29)
[4] Kresse, G.; Hafner, J. Molecular Dynamics for Open-Shell Transition Metals. Phys. Rev. B 1993, 48, 13115−13118.
[5] Blöchl, P. E. Projector Augmented-Wave Method. Phys. Rev. B: Condens. Matter Mater. Phys. 1994, 50, 17953−17979.
[6] Kresse, G.; Joubert, D. From Ultrasoft Pseudopotentials to the Projector Augmented-Wave Method. Phys. Rev. B: Condens. Matter Mater. Phys. 1999, 59, 1758−1775.
[7] Perdew, J. P.; Burke, K.; Ernzerhof, M. Generalized Gradient Approximation Made Simple. Phys. Rev. Lett. 1996, 77, 3865−3868.
[8] Grimme, S.; Antony, J.; Ehrlich, S.; Krieg, H. A Consistent and Accurate Ab Initio Parametrization of Density Functional Dispersion Correction (DFT-D) for the 94 Elements H-Pu. *J. Chem. Phys.* **2010**, *132* (15), 154104. https://doi.org/10.1063/1.3382344.
[9] Grimme, S.; Ehrlich, S.; Goerigk, L. Effect of the Damping Function in Dispersion Corrected Density Functional Theory. *Journal of Computational Chemistry* **2011**, *32* (7), 1456–1465. https://doi.org/10.1002/jcc.21759.
[10] Boo, C.; Chen, B.W.J.; Zhang, J.; Kabiraj, A.; Sullivan, M.B. *CatPlat*: An Automated Heterogeneous Catalysis Platform for High Throughput Screening, 2024.

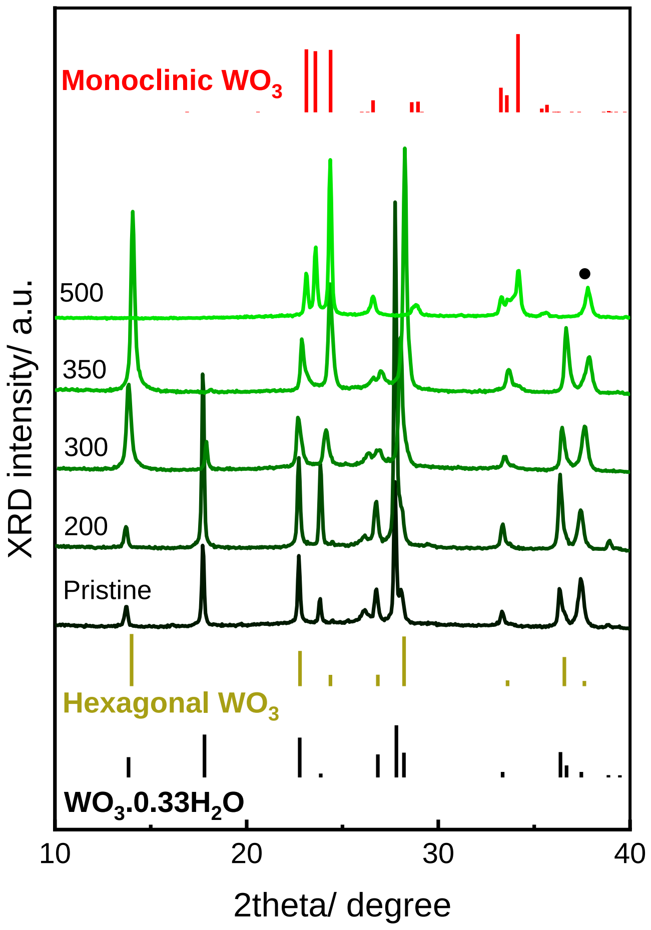

Figure S1 XRD pattern of the as-prepared WO_3 obtained from hydrothermal synthesis and those after being heated at different temperatures (200-500°C) in the air for 2 hours. The black dot (•) indicates the peak of SnO_2 from the FTO substrate.

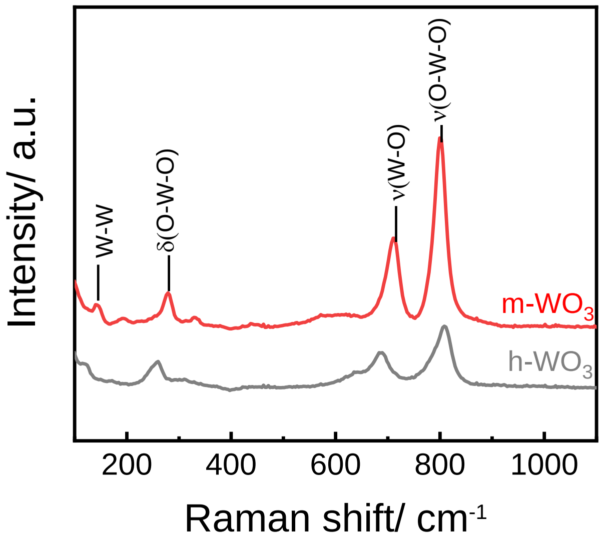

Figure S2 Raman spectra of m-WO_3 and h-WO_3 samples

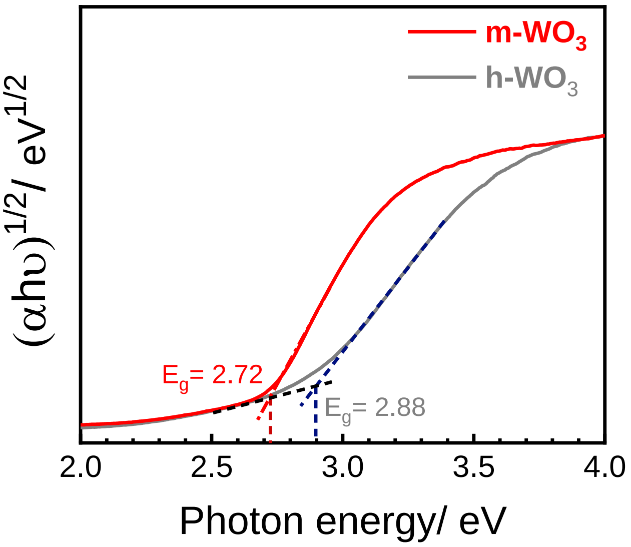

Figure S3 Tauc plot of m-WO_3 and h-WO_3 obtained from their respective UV-Vis spectra.

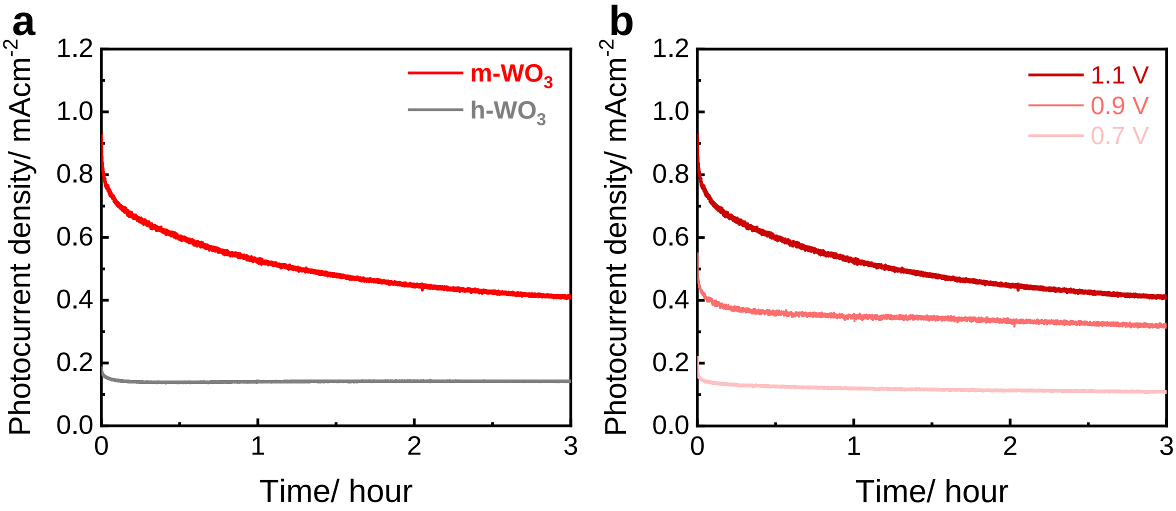

Figure S4 (a) Chronoamperometric response obtained for h-WO_3 and m-WO_3 at 1.1 V_{RHE}; (b) Chronoamperometric response obtained for m-WO_3 at different applied potentials (0.7-1.1 V_{RHE}) in 0.1 M borate electrolyte with 5 mM HMF under AM 1.5G irradiation.

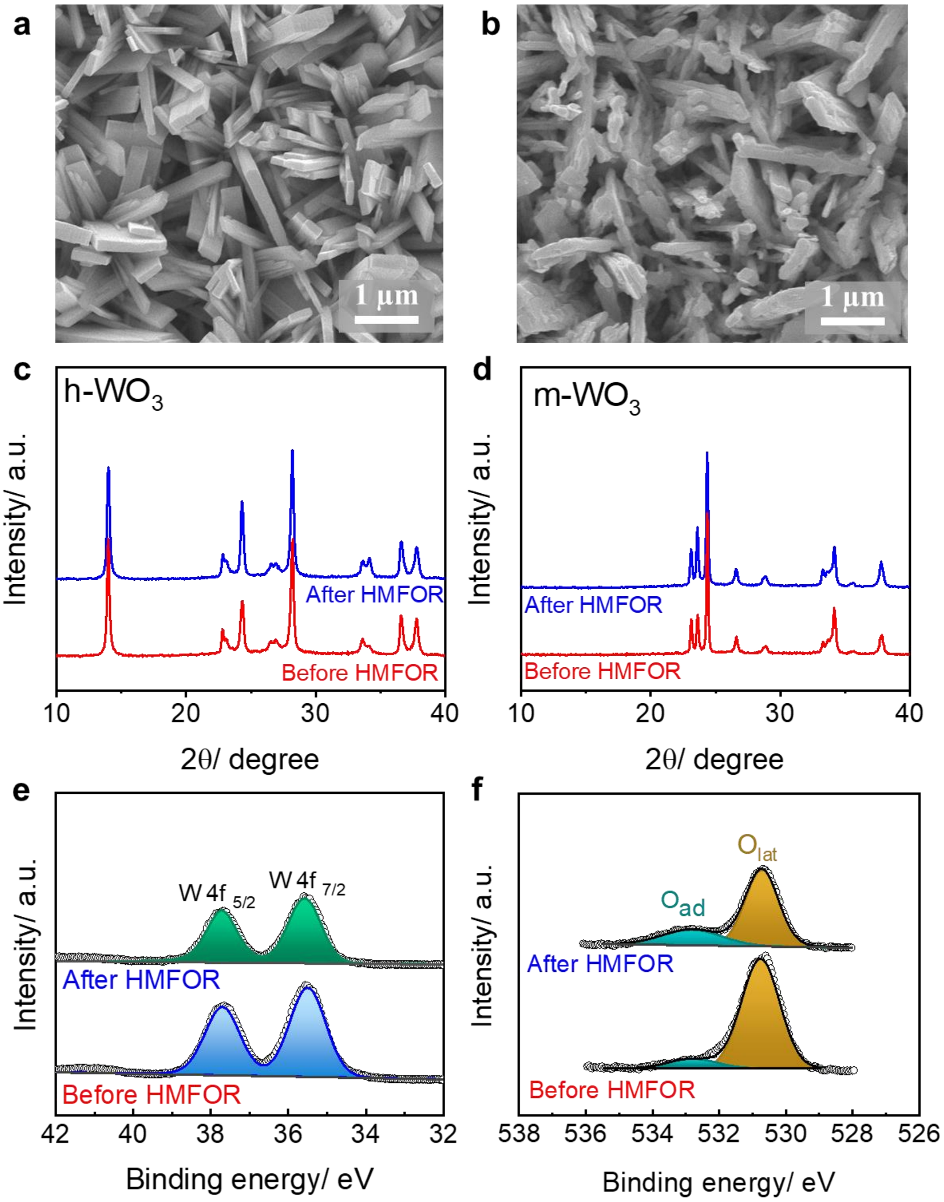

Figure S5 SEM images of (a) h-WO_3 and (b) m-WO_3 after running HMFOR at 1.1 V for 3h; (c, d) XRD spectra and (e, f) XPS W 4f and O 1s spectra of h-WO_3 and m-WO_3 before and after HMFOR.

As shown in **Figures S5a** and **S5b,** the morphology of both samples remained intact, and no destruction was observed. All diffraction peaks detected in the post-HMFOR samples matched perfectly with signals corresponding to initial pristine samples, indicating no phase transition between hexagonal and monoclinic structures under PEC conditions (**Figure S5c,d**). Additionally, the chemical state of W in both h-WO_3 and m-WO_3 samples remained unchanged (**Figure S5**e,f).

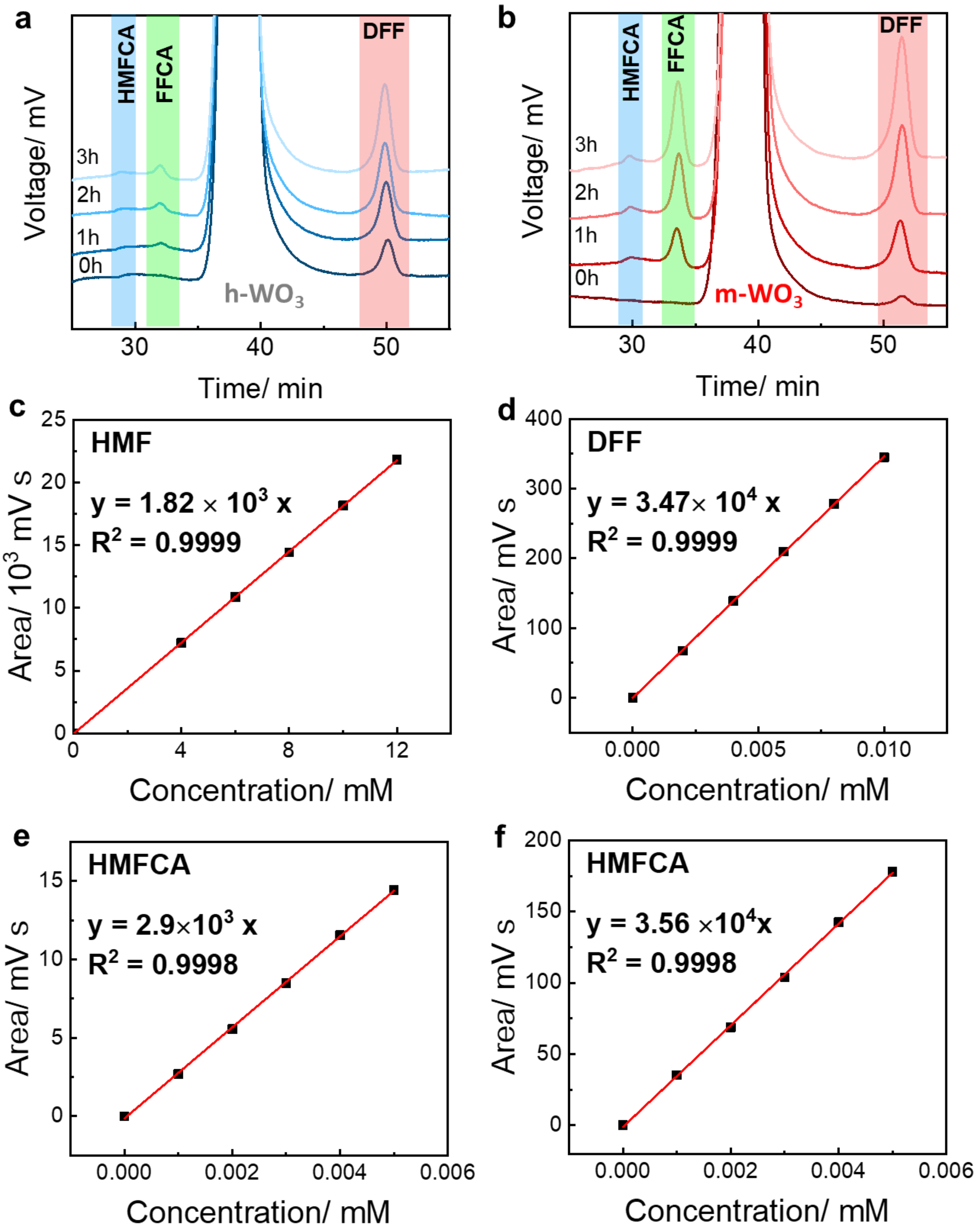

Figure S6 (a, b) Chromatogram obtained from the HPLC analysis of the anolyte collected at hourly intervals during the HMFOR at 1.1 V_{RHE} over (a) h-WO_3 and (b) m-WO_3 photoanodes; (c-f) shows the calibration curves for the corresponding oxidation products.

Table S1 Comparison of PEC HMFOR across different photoanodes.

**Photoanode**	**Electrolyte**	**j (mA cm^{-2})^{a}**	**Product selectivity (%)**	**Ref.**
WO_3	5 mM HMF<br>(NaPi pH 4)	1.52	DFF (NA)<br>FFCA (NA)<br>HMFCA (NA)	*RSC Adv.*, 2021,11, 198-202
$BiVO_4$	5 mM HMF + **TEMPO**[b]<br>(NaBi pH 9)	3.54	FDCA (~100%)	*Appl. Catal B*, 2023, 331,122679
Ti:Fe_2O_3/CoPi	5 mM HMF + **TEMPO**<br>(NaBi pH 9)	0.7	FDCA (90%)	*Sol. RRL*, 2023, 7, 2300205
Fe_2O_3	10 mM HMF<br>(pH 12.5)	0.75	HMFCA (83.9%)<br>DFF (7.4%),<br>FFCA (6.4%)	*Catalysts*, 2021, 11, 969
TiO_2	5 mM HMF + **TEMPO**<br>(NaBi pH 9)	0.7	DFF (11%)<br>HMFCA (8%)<br>FFCA (~55%)<br>FDCA (7%)	*Electrochim Acta*, 2024, 475, 143643
Fe_2O_3/NiOOH	10 mM HMF<br>(pH 12.5)	3.46	FDCA	*Angew. Chem.* 2024, e202413298

[a]: at 1.2 V_{RHE};

[b]: redox mediator.

NA: not available

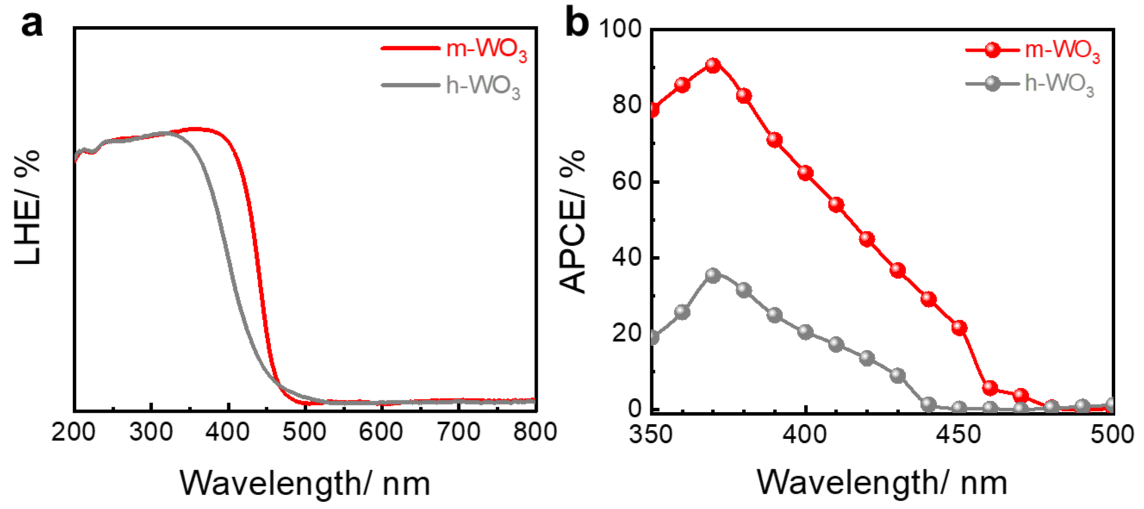

Figure S7 (a) Light harvesting efficiency calculated from the corresponding UV-Vis spectra (b) APCE of h-WO_3 and m-WO_3 photoanodes at 1.1 V_{RHE} in 0.1 M NaBi + 5 mM HMF under AM 1.5G irradiation.

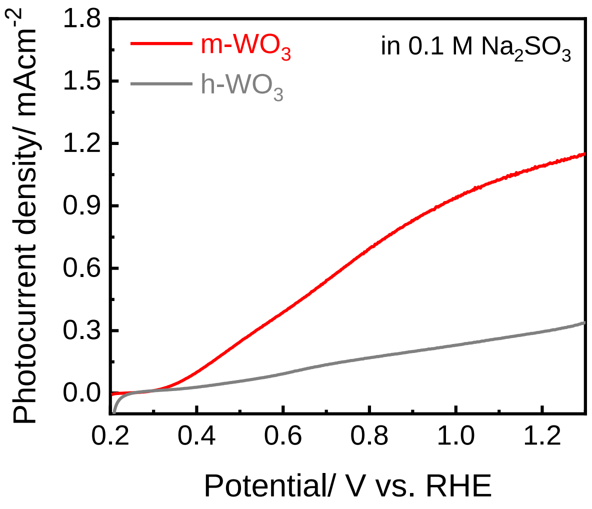

Figure S8 J-V curves of h-WO_3 and m-WO_3 photoanodes in 0.1 M NaBi + 0.1 M Na_2SO_3 (as hole scavenger) under AM 1.5G irradiation.

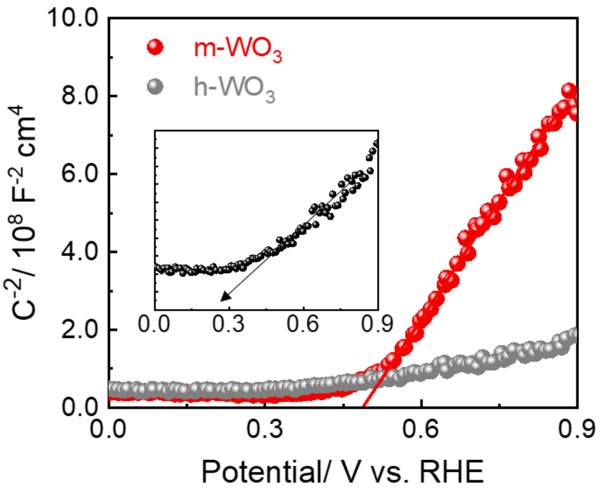

Figure S9 Mott-Schottky plot of h-WO_3 and m-WO_3 photoanodes measured at 1000 Hz in 0.1 M NaBi + 5 mM HMF under dark irradiation

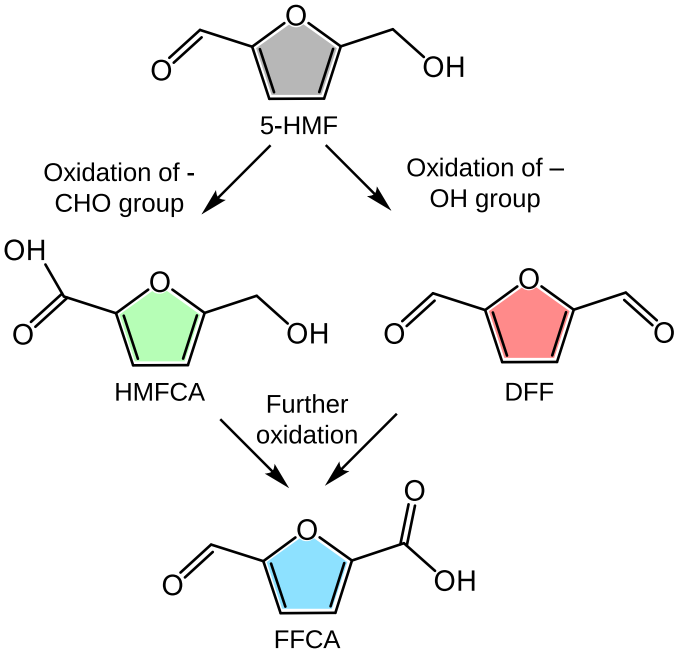

Figure S10 Possible reaction pathway and potential oxidation products from HMFOR

To investigate the preferential oxidation of WO_3 towards DFF and HMFCA, 5 mM of the corresponding chemical was added to the NaBi. The electrochemical setup and conditions were identical to those for PEC HMFOR.

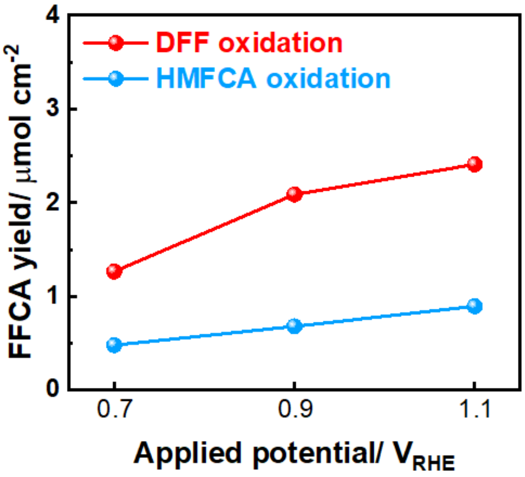

Figure S11 The FFCA yield obtained from the PEC oxidation of DFF and HMFCA under AM 1.5 G irradiation in 0.1 M NaBi + 5 mM DFF or HMFCA over m-WO_3 photoanode.

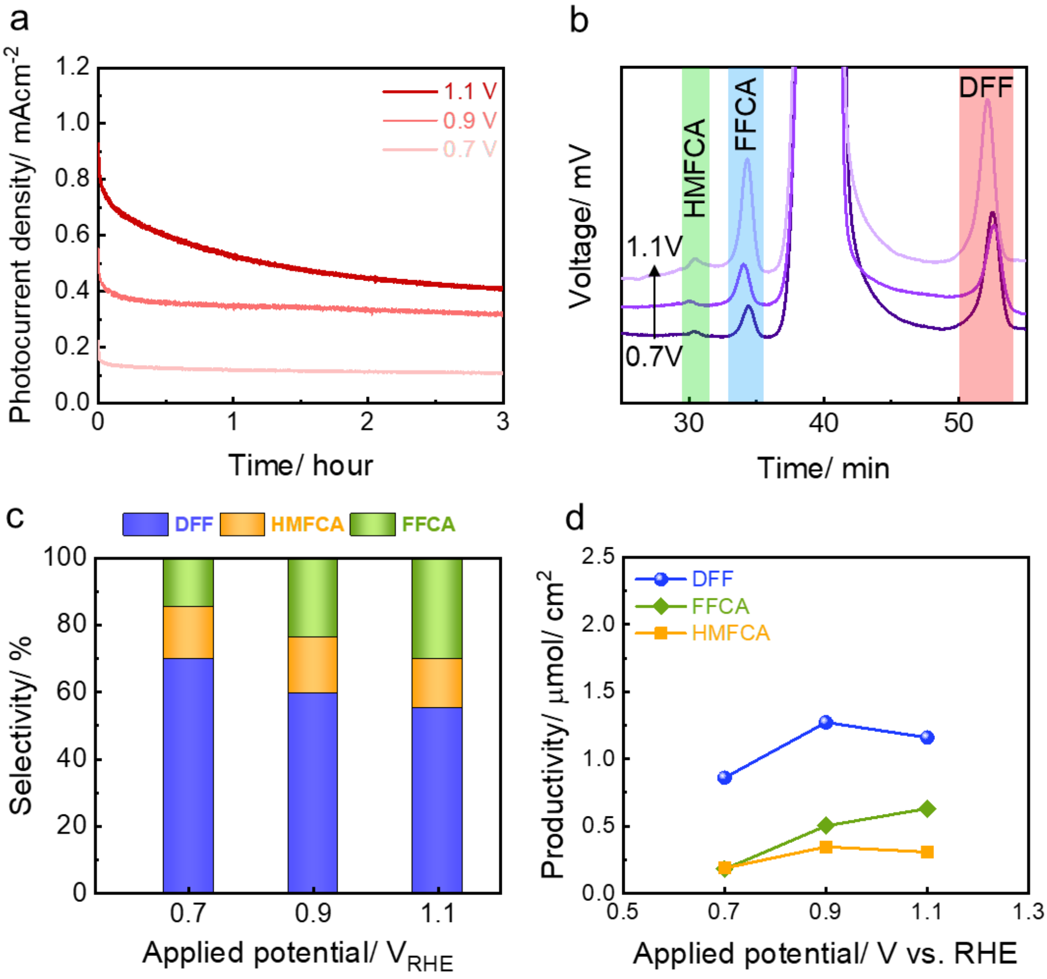

Figure S12 (a) Current-time profile, (b) HPLC chromatogram, (c) Product distribution, and (d) productivity obtained from PEC HMFOR over m-WO_3 at different applied potentials from 0.7 to 1.1 V_{RHE} in 0.1 M Nabi + 5 mM HMF (pH 4) under AM 1.5G irradiation.

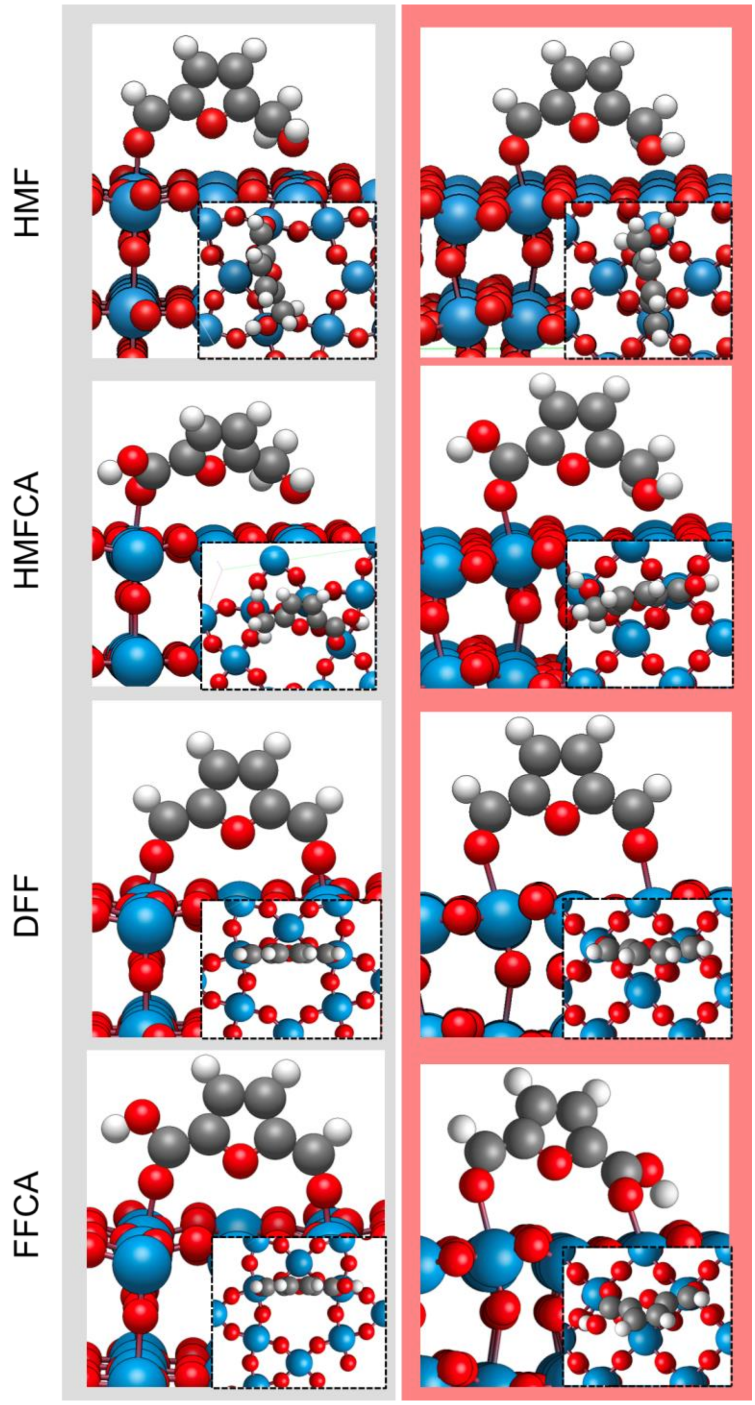

Figure S13 The models of HMF and its oxidized products (HMFCA, DFF, FFCA) adsorbed on the (001) plane of h-WO_3 (first column) and m-WO_3 (second column). The insets show the corresponding side view.
